# “Husa” Sucker

**Published:** 1899-06

**Authors:** 


### "HUSA" SUCKER?

THE "Husa" bubble has been punctured by a Cincinnati chemist and has collapsed. It will be remembered that a Texas doctor, about a year ago, sent a communication to a medical journal announcing the chance discovery of a hitherto unknown and unclassified plant growing in the Florida everglades, and known to the Seminole Indians as husa. It was claimed by him to be a wonderful diffusible stimulant, antidotal to snake poison and an infallible cure of the opium habit. His discovery was given wide publicity and excited general comment. But the exploiter's peculiar evasiveness in responding to requests for specimens of the plant aroused suspicion, and an analysis of his tincture of husa disclosed the fact that it was composed of a large percentage of morphine, with salicylic acid, alcohol and coloring matter. In a word, it was a rank and dangerous fraud.

No blame can be attached to the medical journals which, by publishing accounts of the *quasi* discovery, became unwilling parties to the fraud. It was received by them in good faith and published as an item of considerable professional interest. W.

